# Does Seeing Ice Really Feel Cold? Visual-Thermal Interaction under an Illusory Body-Ownership

**DOI:** 10.1371/journal.pone.0047293

**Published:** 2012-11-07

**Authors:** Shoko Kanaya, Yuka Matsushima, Kazuhiko Yokosawa

**Affiliations:** Department of Psychology, The University of Tokyo, Tokyo, Japan; ICREA-University of Barcelona, Spain

## Abstract

Although visual information seems to affect thermal perception (e.g. red color is associated with heat), previous studies have failed to demonstrate the interaction between visual and thermal senses. However, it has been reported that humans feel an illusory thermal sensation in conjunction with an apparently-thermal visual stimulus placed on a prosthetic hand in the rubber hand illusion (RHI) wherein an individual feels that a prosthetic (rubber) hand belongs to him/her. This study tests the possibility that the ownership of the body surface on which a visual stimulus is placed enhances the likelihood of a visual-thermal interaction. We orthogonally manipulated three variables: induced hand-ownership, visually-presented thermal information, and tactically-presented physical thermal information. Results indicated that the sight of an apparently-thermal object on a rubber hand that is illusorily perceived as one's own hand affects thermal judgments about the object physically touching this hand. This effect was not observed without the RHI. The importance of ownership of a body part that is touched by the visual object on the visual-thermal interaction is discussed.

## Introduction

It has long been assumed that the impact of visual and thermal stimulation is interactive, yet this long-held belief has accumulated surprisingly little experimental support [1]. The most popular and frequently studied version of this view is the Hue-Heat Hypothesis which maintains that light waves, either directly or reflected off surfaces, that have dominant wavelengths near the red end of the spectrum are felt as warm and those toward the blue end of the wavelength spectrum are felt as being cool. However, a number of studies have not supported this hypothesis; consistently, experimental results have shown that any support for such a formulation appears to derive from purely intellectual or cognitive factors, and that hue and colors in general have no significant impact on our actual sensations of thermal heat or comfort. For instance, [2] had participants judge which of two, equally heated, cylinders was warmer when these cylinders were wrapped in papers of different colors; results failed to show any clear effect of colors. Similarly, [3] asked participants to wear goggles with red, blue, or clear lenses and report their thermal comfort in rooms with various levels of air-conditioning. Again, results failed to provide evidence that colors contributed to judgments of thermal comfort.

Recently, however, an intriguing phenomenon has been observed in [4], which employed a modified version of the rubber hand illusion (RHI) [5]. Originally, the RHI is an illusion in which a person feels as if a prosthetic hand made of rubber belongs to him/her. This strange feeling can be induced by touches to a participant's own left (or right) hand occluded from his/her sight, which is synchronized with touches to a prosthetic hand shaped like a left (or right) hand in front of him/her. Notably asynchronous touches do not induce this illusion [5]–[7]. The main finding of [4] was that an illusory hand-ownership similar to that described above can also be obtained by merely the sight of a laser light stroking a prosthetic hand positioned near one's own unseen hand (i.e., in the absence of any tactile stimulation to the hidden real hand). Another notable observation in that situation was that participants felt illusory thermal (or tactile) sensations to their own hand when the prosthetic hand was struck by the laser light. This experiment was designed primarily to demonstrate the existence of a previously unknown illusion concerned with body ownership itself, so [4] neither did rigorously measure participant's thermal judgments nor did this study manipulate the actual temperature of the stimulus (a laser light). Nevertheless, this report was the first to successfully demonstrate evidence of a vivid visual-thermal interaction.

Accordingly, it is useful to pursue this phenomenon with the aim of identifying its determining factors. Specifically, it is important to differentiate factors involved in creating visual-thermal interactions, such as that observed in [4], from factors that resulted in no effects, such as those reported in other previous studies [1]–[3]. A possible explanation for the visual-thermal interactions observed in [4] involves the perceptual ownership of the body surface on which the visual stimuli had contact; this sensation was emphasized in the procedure of [4] by providing participants with the impression that the prosthetic hand in front of them was their own. This type of manipulation was not employed in other studies. Therefore, it is possible that visual information can affect a participant's thermal judgment of a stimulus, but this only happens when the body surface involved is experienced as belonging to an individual's own body when touched by this visual object. Another explanation involves the role of “apparently-thermal” visual stimuli. Perhaps in our daily lives, the familiarity with a particular type of visual stimulation is linked to a specific temperature (e.g. the sight of an ice cube is a valid cue for coldness); if this is the case, then certain visual stimuli may simply be more likely to elicit specific temperature responses than others. In [4], a red-colored laser light was used, and this might be more likely to give an impression of warmth or heat than colored surfaces, such as papers or lenses [2], [3]. It is possible that thermal information conveyed visually is weighted more heavily when realistic stimuli are involved assuming that our knowledge about everyday experiences is reflected into the sensory system.

The influence of visual information on thermal perception is predicted in light of another line of research. It has been shown that the thermal perception is regulated not only by the physical thermal level of stimuli but it also appears to interact with other perceptual or cognitive processes. This is evident in an illusion, termed “thermal referral” [8], [9], which is induced by concurrent touches with some objects heated differently. If an observer touches three different thermal stimulators simultaneously using their middle three fingers when only the two outer fingers are stimulated by warm (or cool) stimuli, the central finger (receiving a neutral temperature) will also feel warm (or cool). Besides this, tactile sensation also modulates the perceptual quality of thermal stimuli [10], [11]. It has also been reported that the “thermal-grill” phenomenon, which involves illusory pain that is caused by unusual thermal stimulation, can be mediated by higher-order body representation [12]. Taken together, these reports support the idea that thermal perception is substantially influenced by other components of our sensory system; in turn, this suggests the possibility of visual-thermal interaction.

In the present study, we investigated the relationship between a putative visual-thermal interaction and perceptual body-ownership. We hypothesized that the sight of a visually-thermal object touching the body surface can affect our thermal judgment only when we consciously perceive relevant body surface as belonging to ourselves. To pursue this hypothesis, we independently manipulated modes of stimulus presentation (visual, tactile) of thermal information with a means of inducing body ownership (synchronous, asynchronous presentations). In addition, the visual presentations of thermal information was realistic, hence likely to induce a specific thermal sensation in real life, here coldness (e.g. an ice cube). In order to manipulate hand-ownership, we enlisted the original version of the RHI [5]. This involved presenting two different blocks of trials in which one block featured induction of an illusory ownership of a prosthetic hand (“rubber hand”) by touches to one of the participant's own hand (“real hand”) synchronized with touches to the rubber hand. This condition was predicted to induce illusory ownership of the rubber hand. However, in the other block, the illusory hand-ownership was not expected because touches to real and rubber hands were asynchronous. In each of the two blocks, the participant performed a thermal change judgment task immediately following the RHI induction phase (with synchronous or asynchronous touches). The thermal judgment tasks consisted of presenting two pairs of stimuli consecutively. In the first pair (“presentation 1”), one object of a specific temperature was placed on the real hand (e.g., plastic cube) and the other object visually indicating a specific temperature (e.g. ice cube) was placed on the rubber hand. This was immediately followed by a second pair of objects. That is, the tactile thermal stimuli were always placed on the real hand, while the visual thermal stimuli were always placed on the rubber hand, independently. The thermal properties of the two stimuli successively presented to a given hand were sometimes identical and sometimes different physically (on the real hand) or visually (on the rubber hand). A participant's task was to judge whether the physical temperature of the object on the real hand had risen or dropped on presentation 2 relative to its temperature on presentation 1. Note that this is a thermal change discrimination task, presumably based purely on the perception of the relative physical temperature of the object placed on the real hand. If visual information of an object presented to the rubber hand influences participants' thermal judgments, then their reports should show a systematic impact of this in their thermal judgments of objects presented to the real hand counterpart. In this case, we should be able to infer that an individual's experience of the object's temperature is affected by visual information associated with perception of the tactile object. Furthermore, if the conscious ownership of body parts is critical to visual-thermal interaction, then this effect should be observed only in the block where touches are synchronized over the two hands.

## Methods

### Ethical Statement

Participants gave verbal informed consent, which was recorded and kept by the experimenter. The verbal consent was approved by the ethical committee of Department of Psychology, the University of Tokyo, since participants were undergraduate students attending a psychology class there and they had firmly understood the general procedure of psychological experiments and the privacy policy. All of the other procedures described below were approved by the same ethical committee, and conducted according to the principles and guidelines of the Declaration of Helsinki.

### Participants

A total of 20 participants including 8 male and 12 female university student aged from 21 to 23 participated in this experiment. They were neurologically healthy and had normal or corrected-to-normal vision. The temperature of the surface of their left hand measured before the experiment was normal with a mean of 29.59 degrees (centigrade).

### Configuration of Experiment

**Figure 1:**
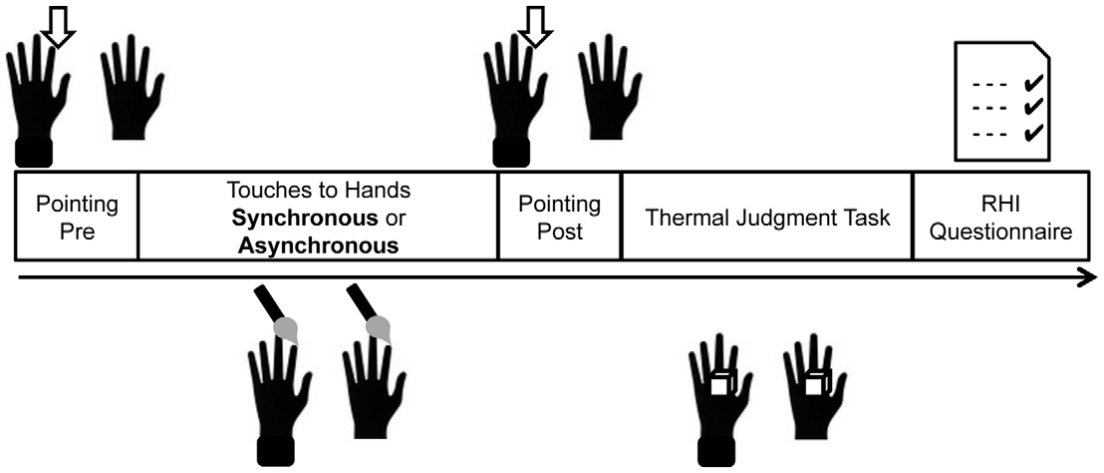
Procedure. The experimental procedure in one block. The hand on the left side in each icon represents participants' own left hand (“real hand”) ; the one on the right side represents the prosthetic hand (“rubber hand”).

The experiment consisted of two blocks. The configuration of procedures within a single block is shown in Figure 1. Each block involved a RHI-induction phase followed by a thermal change judgment task. Specifically, the RHI induction phase involved touches to the two hands (real and rubber hands), pointing tasks before and after the touches, and the questionnaire. Two blocks differed with respect to synchronicity of touches. In the synchronous-touch block, touches were always given simultaneously to the two hands. In the asynchronous-touch block, they were given alternately. All other procedures were identical in the two blocks. The block order was counterbalanced.

### Apparatus and Stimuli

**Figure 2:**
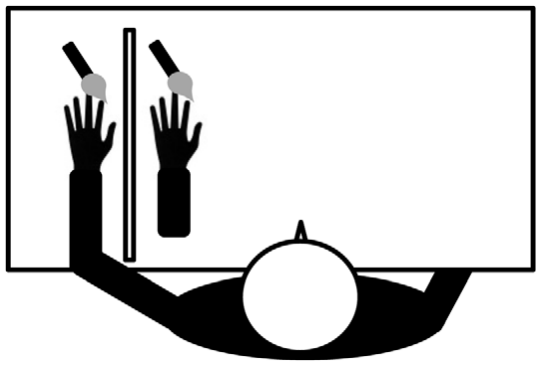
Experimental set-up. The schematic illustration of a participant sitting in front of the rubber hand with one real hand hidden from sight by a partition. These two hands are stimulated by identical two paint brushes.

Participants sat resting their left arm on a marked place on the table; hereafter this is referred to as the “real hand”. Then, a partition was positioned to hide this real hand from the participant's view. A life-sized rubber model of a left hand (“rubber hand”), was placed on the table in front of the participant, parallel to the real hand (Figure 2). Participants were told to keep their right hand, which is irrelevant to the RHI, hidden from view under the table. They were also told not to move this hand except when performing pointing tasks in the RHI induction phase. To administer touches to these two hands, two identical paint brushes were used.

In the thermal change judgment task, “neutral (N)” or “cool (C)” stimulus objects were presented respectively to each of the two hands. On the real hand, these were provided as tactile (T) stimuli with certain temperatures. A plastic cube heated to stable 31 degrees (centigrade) served as a tactile neutral (TN) stimulus; another plastic cube heated to stable 22 degrees (centigrade) served as a tactile cool (TC) stimulus. On the rubber hand, objects were provided as visual (V) stimuli that indicated a specific temperature only by its appearance. A plastic cube at the room temperature (without any modification of its physical temperature) served as a visual neutral (VN) stimulus, or a piece of ice served as a visual cool (VC) stimulus. These plastic cubes were 25 millimeters on a side and were translucent. The ice cube was approximately the same size as the plastic cubes.

### Design and Conditions

First, we manipulated the synchronicity of touches to two hands in the RHI induction phase as a between-block factor. It has been reported that the RHI is induced by synchronous touches to the rubber and real hands, but not by asynchronous touches to them [5]–[7]. Accordingly, we hypothesized that the RHI would be induced in the synchronous-touch block, but not in the asynchronous-touch block. Two indices of the strength of the RHI are the proprioceptive drift and the RHI questionnaire, both of which have been used in related studies. It has been reported that participants' subjective localization of their own hand is often misperceived or shifted in the direction of the rubber hand under the RHI, and that proprioceptive drift is a measure of this shift in the direction of the rubber hand [5], [7], [13]. The RHI questionnaire included questions used by [5] that were translated into Japanese.

In the thermal change judgment task, we orthogonally manipulated two variables within each block: Four object change types on the real hand from the presentation 1 to 2 were crossed with four object change types on the rubber hand, creating 16 conditions. Generally, on each hand, the objects were replaced to either create a thermal change (N to C, C to N) or not (N to N, C to C); on the real hand the four types of tactile changes were: TN to TC, TC to TN, TN to TN, TC to TC; on the rubber hand the four types of visual changes were: VN to VC, VC to VN, VN to VN, VC to VC. Participants underwent one trial in each of these 16 conditions with a total of 16 trials in a randomized order.

### Procedure

Within each block, the experimental procedure was as follows. At first, participants were asked to point (with the tip of the right index finger) to a position on the underside of the table to indicate the location at which they felt to be just under the tip of the left index finger (of the real hand). The experimenter recorded this position for a comparison with that after visual-tactile touches.

Next the experimenter started to give rhythmic touches to the same spatial locations on the real and rubber hands using two paint brushes. These visual-tactile stimulations were given synchronously to them in the synchronous-touch block, while they were given one after another in the asynchronous-touch block, while the total number of touches was equal between these two blocks. Touches were given for 10 seconds per one position randomly selected from 6 candidate positions, including three joints on the index and middle finger of both hands. The total duration of stimulation was 10 minutes.

At the end of this stimulation, we again asked participants to point to the position they felt just under the left index finger. The disparity between indicated positions before and after the stimulation was the proprioceptive drift [5].

**Figure 3:**
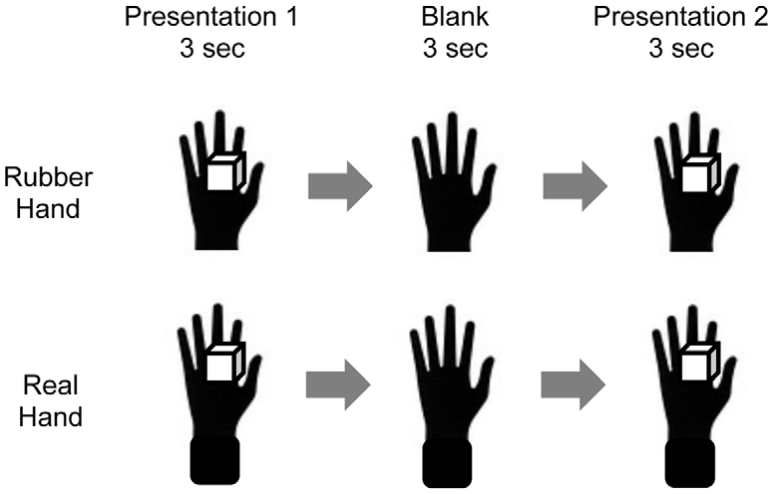
Stimulus presentation of thermal change judgment task. Stimulus presentations of the thermal change judgment task in one trial. Pairs of visual and thermal stimuli are presented to the real and rubber hands respectively, before (presentation 1) and after (presentation 2) the blank.

Next, the 16 trials of the thermal change judgment task were performed. On each trial, the experimenter presented a pair of objects (presentation 1) to real and rubber hands respectively at the same time for 3 seconds (Figure 3). This was repeated again (presentation 2) after a blank of 3 seconds. During these two presentations, the types of objects presented to each hand were independently determined (see above). On each trial, following presentation 2, we asked participants to report verbally whether they felt the temperature of the object on the real hand to have risen or dropped relative to presentation 1.

Finally, we had participants complete the RHI questionnaire. It included nine items describing subjective experiences (Table 1). According to [5], items 1, 2 and 3 are assumed to relate to the RHI. Participants recalled their experiences under the RHI induction phase and indicated their agreement with each item on a seven-step scale from 1 to 7, in which 1 means a strong disagreement, 4 is neutral and 7 means a strong agreement.

**Table 1:** Rubber hand illusion questionnaire.

Number	Question
1	It seemed as if I felt the touch of the paintbrush in the location where I saw the rubber hand touched.
2	It seemed as though the touch I felt was caused by the paintbrush touching the rubber hand.
3	I felt as if the rubber hand were my hand.
4	It felt as if my (real) hand was drifting towards the right (towards the rubber hand).
5	It seemed as if I might have more than one left hand or arm.
6	It seemed as if the touch I was feeling came from somewhere between my own hand and the rubber hand.
7	It felt as if my (real) hand was turning “rubbery”.
8	It appeared (visually) as if the rubber hand was drifting towards the left (towards my hand).
9	The rubber hand began to resemble my own (real) hand, in terms of shape, skin tone, freckles or other visual features.

The rubber hand illusion questionnaire initially used in Botvinick & Cohen (1998). We translated this into Japanese.

## Results

**Figure 4:**
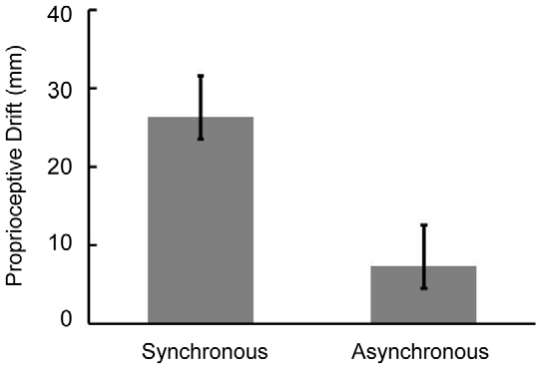
Results of proprioceptive drift. Mean proprioceptive drift, an index of the RHI, as function of synchronous-touch and asynchronous-touch blocks. Positive value of the drift index means that the participant mis-localized his/her own hand in the direction of the rubber hand after visual-tactile stimulation.

In order to ascertain whether an RHI had actually occurred in the synchronous-touch block and not in the asynchronous-touch block, we compared the amount of proprioceptive drift and ratings in the questionnaire between these two blocks. Figure 4 shows the mean value of the proprioceptive drift in each block. This is calculated as the shift of the indicated positions of the participant's left index finger toward the direction of the prosthetic hand after the exposure to touches; a positive value means that the indicated position shifted toward the rubber hand. A paired t-test revealed a significantly larger proprioceptive drift in the synchronous-touch block than that in the asynchronous-touch block (*t* (19)  = 3.76, *p*<.05). This means that the RHI occurred only with synchronous touches to two hands.

**Figure 5:**
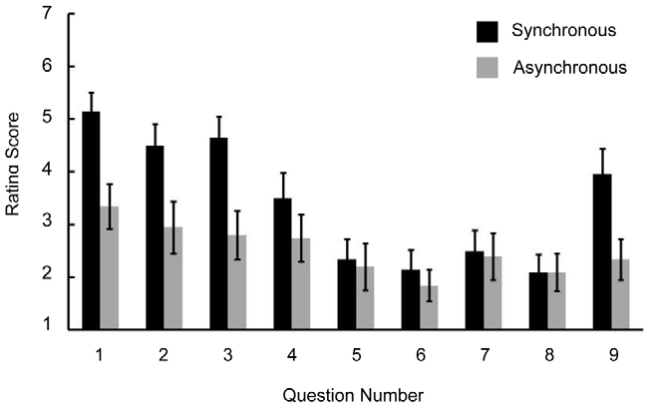
Results of rubber hand illusion questionnaire. Results from the rubber hand illusion questionnaire. Participants rated their agreement to each question on a 7 point scale.

The averaged ratings on the questionnaire are shown in Figure 5. A repeated-measures ANOVA with 2 factors as block condition (synchronous-touch and asynchronous-touch) and item on the questionnaire (from item 1 to item 9) revealed a significant main effect of block condition, questionnaire item and an interaction (*F* (1,19)  = 13.98, *p*<.05, *F* (8,152)  = 11.66, *p*<.05, *F* (8,152)  = 3.74, *p*<.05, respectively). Ratings on item 1–3 and 9 in the synchronous-touch block were higher than those in the asynchronous-touch block (*p*<.05). This trend is similar to results from previous studies that employed the same questionnaire [5], [14].

**Figure 6:**
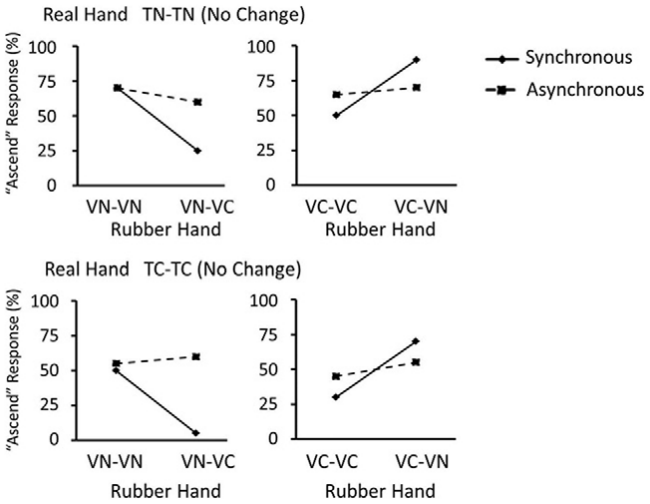
Results of thermal change judgment task: no change on the real hand. Results from the thermal change judgment task on trials with no change on the real hand when the object on the real hand is neutral (A) and cool (B). On the ordinate is percent of ‘ascend’ responses on the real hand.

**Figure 7:**
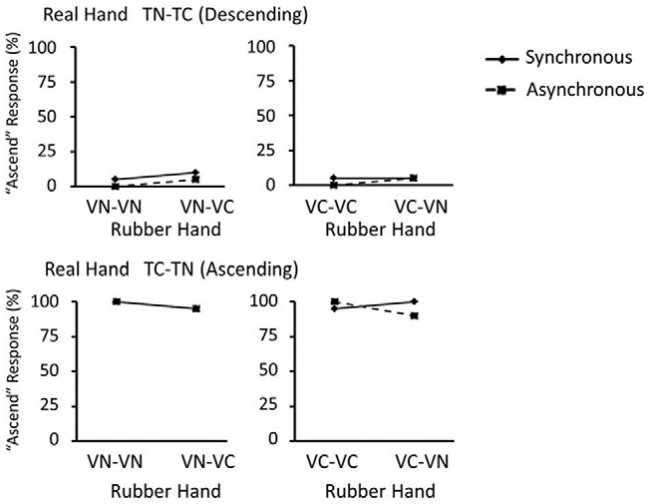
Results of thermal change judgment task: change on the real hand. Results from the thermal change judgment task on trials with changed temperature on the real hand when it decreases (A) and increases (B). On the ordinate is percent of ‘ascend’ responses on the real hand.

To pursue results in the thermal judgment task in each block, firstly we broke 16 conditions into 8 pairs, each comprising two conditions to be compared. In each pair, two conditions have the same change type on the real hand, and the same object on the rubber hand in the presentation 1. However, they differed in the object on the rubber hand in the presentation 2. Then, the proportion of participants who reported that the object temperature on the real hand ascended from the presentation 1 to the presentation 2 (‘ascend’ response) was compared within each pair. For example, we assessed whether or not the proportion of ‘ascend’ responses was significantly different between the TN-TN × VN-VN condition and the TN-TN × VN-VC condition (i.e., only the second object on the rubber hand differed between these two conditions). This analysis was performed on data separately for the synchronous-touch block and the asynchronous-touch block. Figures 6 and 7 show data in all pairs subjected to this analysis in both of the two blocks. The McNemar test revealed that significant differences were found in proportion of ‘ascend’ responses in four pairs where the object did not change on the real hand (TN-TN, or TC-TC) but it did change on the rubber hand (VN-VC, or VC-VN), in the synchronous-touch block, including TN-TN × VN-VC condition (*χ* ^2^ = 5.40, *p*<0.05), TN-TN × VC-VN condition (*χ* ^2^ = 8.00, *p*<0.05), TC-TC × VN-VC condition (*χ* ^2^ = 9.00, *p*<0.05), and TC-TC × VC-VN condition (*χ* ^2^ = 4.57, *p*<0.05). Other pairs in the synchronous-touch condition and all pairs in the asynchronous-touch condition did not show any significant differences.

We conducted further analyses to statistically compare the results in the synchronous-touch block with those in the asynchronous-touch block. For conditions in which object physically changed on the real hand, we broke down these trials into four groups on the basis of the synchronicity of touches and the presence of the object changes on the rubber hand. Then we calculated the ratio of responses which were in accordance with the direction of changes (e.g. ‘ascend’ responses were in accordance with VC-VN changes and ‘descend’ responses were with VN-VC changes) for each group. The resulting index, termed “visually-dominated response ratio”, was provided per participant. The averaged ratio, taken over all participants, is shown in the left half of Table 2. A 2×2 repeated measures ANOVA with factors of RHI induction condition (synchronous-touch, asynchronous-touch) and tactile changes on real hand (presence, absence) was applied to these response ratio scores. Results indicated the presence of significant interaction between these two factors (*F* (1,19)  = 32.02, *p*<.05). This reveals that in the absence of tactile changes to the real hand, the ratio was significantly higher in the synchronous-touch block than that in the asynchronous-touch block. Indeed, the response ratio value was significantly above chance level only in the one of the four conditions. This condition is the one involving synchronous-touches and no tactile change to the real hand (*t* (19)  = 7.44, *p*<.05). Subsequently, we conducted a similar analysis for trials in which there was no change on the rubber hand. The response ratio value in this case was calculated as the ratio of trials in which participants' responses were in accordance with the “sign” of the second object on the rubber hand. The term “sign” here indicates the relative warmth of the object (positive for the neutral object and negative for the cool object), so ‘ascend’ responses were in accordance with VN-VN and ‘descend’ responses were with VC-VC). The average for this ratio is shown in the right half of the Table 2. As the two stimulus objects were presented consecutively in this thermal change judgment task, it is possible that the second (more recently experienced) object on the rubber hand has a stronger influence on judgments. For instance, people may not correctly remember the physical thermal property of the first object. If this is happening, then the sign of the second object on the rubber hand should have a significant effect on results even when the object on the rubber hand doesn't change. However, a two-way repeated measures ANOVA with the touch synchrony (synchrony, asynchrony) and thermal change (presence, absence) failed to show any main effects or an interaction over these trials. Also, none of these values differed from chance levels.

**Table 2:** Visually-dominated response rates in the thermal change judgment task.

Rubber hand	Change		No Change	
Real hand	Change	No change	Change	No change
Synchronous- touch	0.49 (0.15)	0.83 (0.20)	0.5 (0.08)	0.59 (0.23)
Asynchronous- touch	0.51 (0.10)	0.49 (0.20)	0.53 (0.08)	0.54 (0.20)

Mean visually-dominated response rated in each condition groups with standard deviations in parentheses.

## Discussion

We found that thermal judgments about an object placed upon an individual's hand are modified by the sight of an object that touches a nearby prosthetic hand when this individual perceives the latter hand as his/her own. Both the proprioceptive drift and the RHI questionnaire ratings showed that participants experienced a typical RHI in the synchronous-touch block but not in the asynchronous-touch block. Under this illusion, the rubber hand is perceived as one's own hand, and an object on the surface of the rubber hand is also perceived to be touching one's own hand. Participants' subjective reports on the questionnaire confirmed this view in that the rating scores on the questionnaire items, “It seemed as if I were feeling the touch of the paintbrush at the same location where I saw the rubber hand touched”, and “It seemed as though the touch I felt was caused by the paintbrush touching the rubber hand”, were significantly higher in the synchronous-touch block.

In the thermal change judgment task, participants reported the temperature change on objects that touched their real hands during two successive presentations in which objects presented to real and rubber hands were independently manipulated. Firstly we compared the proportion of participants reporting ‘ascending’ changes in a condition where the objects do not change on the rubber hand with a corresponding proportion in the condition where these objects changed; the two successive objects on the real hand and the first object on the rubber hand were held constant for this comparison. In the synchronous-touch block, we found a significant decrease or increase in the proportion of ‘ascend’ responses for the condition in which the apparent temperature of objects placed on the rubber hand dropped (plastic cube to ice) or rose (ice to plastic cube), when compared to conditions in which no such change occurred. Secondly, we found that the proportion of trials on which participants reported a temperature change consistent with the object change on the rubber hand was statistically higher in the synchronous-touch block than the corresponding proportion observed for the asynchronous-touch block, when the physical temperature of the object on the real hand doesn't change. Participants responded in a way that revealed a significant influence of the thermal appearance of objects placed upon the rubber hand; however, this influence was only apparent when participants were vulnerable to the illusory body ownership of the rubber hand and when the actual temperature change of the object on the real hand was not provided.

An intuitive belief that visual appearance of an object (mainly its color) should have some influence on our thermal perception has been summarized in the “Hue-Heat Hypothesis.” However, this notion was called into question by a number of previous studies [1]–[3]; nevertheless, one study [4] has reported that participants can experience an illusory heat of a laser light on a prosthetic hand in conjunction with perceived ownership of the hand. In the current study, we hypothesized that the visual-thermal interaction, suggested by [4], was elicited by a highlighting of the participants' ownership of the prosthetic hand in that procedure. Our present findings confirm this hypothesis. In our thermal change judgment task, the object change on the rubber hand affected participants' thermal judgment significantly, but importantly this happened only when the hand on which the visual object touched was felt as their own hand under the RHI. That is, this happened only in the synchronous condition.

It is also possible that the use of realistic, “apparently-thermal” visual stimuli contributed to this effect. [4] used a red-colored laser light, which could possibly be interpreted as giving out some heat, whereas previous studies which failed to find an effect used simple colored surfaces such as papers or lenses [2]–[3]. Here, we too applied more realistic stimuli, ones that would be naturally evocative of a thermal experience. Specifically, we used an ice cube to convey coldness versus a neutral plastic cube presumably suggestive of ordinary temperature. This idea is that an ecological association between a specific visual stimulation and a corresponding thermal experience in our natural environment, (i.e., rather than just colors) may exert a strong influence on thermal perception. Nevertheless, the effect of color cannot be dismissed completely, since [4] has reported that their informal observation suggested that the use of bluish laser light brought a different effect from that of a red laser.

Participants' thermal judgments on trials in which the temperature of the object presented to a real hand actually rose or declined was not affected by the change on the rubber hand, regardless of whether the RHI was present or not. Instead, their judgments of actual changes were fairly accurate. This can be ascribed to a ceiling effect due to our use of easily discriminable temperatures, i.e., 22 and 31 degrees centigrade. Seemingly, visual effects on our thermal judgment were present only when the actual temperature on the real hand didn't change, hence this thermal information can be considered as ambiguous. Visual information can be utilized as another reliable source of thermal information for an object when physical thermal information is vague and unreliable. This interpretation is in accordance with a common principle of multi-sensory integration; information from one sensory modality has effects on another modality when the former is reliable and the latter is less reliable, when the two sensations are taken to specify the same object or event [15], [16].

Finally, our findings are also relevant with respect to properties of the RHI. The boundary of our bodily self, altered by just a brief exposure to an unusual correlation of visual and tactile stimulation, has been a central topic of psychological and neurophysiological investigations. It has been shown that the RHI is not just a subjective experience of an alien hand as one's own. Rather, it includes a broad range of modification of one's body representation [6], [17]–[19]. [20] have found that threatening the prosthetic hand under the RHI induces strong activity in cortical and sub-cortical areas that are related to anxiety or the urge to withdraw the hand to escape from the threat. They concluded that the prosthetic hand is fully incorporated into a central representation of the body. Our finding lends support to this view because thermal sensation, which plays a substantial role in interoceptive system, was shown to be affected by the RHI. On the other hand, it has been reported that the RHI does not modulate other sensory qualities of the touched object such as roughness [21]. They point out that effects of the RHI on the perception of roughness can be dissociable from other consequences in that it includes a cognitive interpretation of the surface information, at a higher stage. Furthermore, the impact of RHI on motor control of the arm remains a controversial topic [22]. More specification of the scope of our body representation which is subject to the RHI would be needed.
